# Microbial metabolites and MAMPs: orchestrators of intestinal stem cell function

**DOI:** 10.1080/19490976.2026.2755115

**Published:** 2026-11-04

**Authors:** Sudhanshu Shekhar, Dagmar Šrůtková, Martin Schwarzer

**Affiliations:** a Laboratory of Gnotobiology, Institute of Microbiology of the Czech Academy of Sciences, Novy Hradek, Czech Republic

**Keywords:** Microbiota, signals, metabolites, MAMPs, intestinal stem cells

## Abstract

Intestinal stem cells (ISCs) are essential for maintaining and regenerating the intestinal epithelium. Recent studies have highlighted the critical role of the gut microbiota in regulating ISC function through diverse molecular signals, particularly microbial metabolites and microbe-associated molecular patterns (MAMPs). Microbial metabolites, such as short-chain fatty acids (SCFAs) and indoles, modulate ISC activities by influencing proliferation, differentiation, and overall epithelial renewal. MAMPs, such as lipopolysaccharide (LPS) and peptidoglycan-derived muramyl dipeptide (MDP), interact with pattern recognition receptors (PRRs), including Toll-like receptors (TLRs) and nucleotide-binding oligomerization domain (NOD)-like receptors (NLRs), affecting ISC proliferation and epithelial regeneration. In this review, we discuss emerging evidence on how these microbial signals regulate ISC function and elucidate the mechanisms underlying epithelial renewal and tissue repair. A comprehensive understanding of these host-microbe interactions holds therapeutic potential for enhancing epithelial regeneration and restoring gut homeostasis, paving the way for novel regenerative medicine strategies.

## Introduction

The intestinal epithelium is essential for gut homeostasis owing to its ability to absorb nutrients and water, form a physical and biochemical barrier against pathogens, and regulate immunity.^1^ ^,^ ^2^ In adults, this epithelium undergoes renewal every 3-5 days, a process facilitated by intestinal stem cells (ISCs), which are crucial for maintaining epithelial integrity and enabling tissue repair.^3^ ^,^ ^4^ ISCs produce progenitor cells that subsequently develop into different types of specialized epithelial cells, such as enterocytes, goblet cells, enteroendocrine cells, and Paneth cells, to perform the function of sustaining the epithelial integrity.^3^ ^,^ ^5^ A combination of intrinsic genetic programs and extrinsic signals from the surrounding micromilieu, including cytokines, growth factors, and commensal microbes, controls the activity and fate of ISCs.^6^ Growing evidence indicates that the gut microbiota profoundly influences ISC biology via a diverse range of microbial signals. These include microbial metabolites and microbe-associated molecular patterns (MAMPs), which influence epithelial cell proliferation, differentiation, and regeneration by modulating intrinsic signaling pathways essential for ISC maintenance and function,^7^ ^,^ ^6^ Microbial metabolites, such as short-chain fatty acids (SCFAs), lactate, and indoles, influence ISC function by modulating both proliferation and lineage commitment.^8-10^ ^,^ ^11^ On the other hand, MAMPs, such as lipopolysaccharide (LPS) and peptidoglycan-derived muramyl dipeptide (MDP), interact with their cognate pattern recognition receptors (PRRs), such as Toll-like receptors (TLRs) and nucleotide-binding oligomerization domain (NOD)-like receptors (NLRs), which are expressed either on the surface or within ISCs and/or their niche cells.^12^ ^,^ ^13^ These microbial cues activate downstream molecular pathways to modulate ISC function and fate decisions.^11^ ^,^ ^14-16^

This review discusses the contemporary understanding of microbial regulation of ISCs, underscoring the mechanistic pathways by which microbiota-derived signals assimilate into host signaling networks to orchestrate intestinal epithelial homeostasis and repair. A profound knowledge of these processes could result in innovative therapeutic approaches to enhance intestinal regeneration and restore epithelial integrity in clinical conditions, such as inflammatory bowel disease (IBD) and epithelial injury.

## ISC and its niche

The intestinal epithelium consists of two main compartments: the villi and crypts of Lieberkühn (commonly called crypts). Villi are projections that resemble fingers, formed by fully differentiated epithelial cells, such as enterocytes, goblet cells, and enteroendocrine cells, which develop in the crypts and ascend^5^ (Figure 1). These cells are discarded at the apex of the villus as a result of anoikis, a type of programmed cell death enabled by the loss of cell-surrounding extracellular matrix adhesion.^17^ In contrast, the crypt niche harbors a proliferative compartment that encompasses ISCs. These ISCs are characterized as crypt base columnar (CBC) cells, which are actively cycling, long-lived, and multipotent. Additionally, they express the marker leucine-rich repeat-containing G protein-coupled receptor 5 (Lgr5), also referred to as Gpr49. Lgr5 functions as a receptor for R-spondins, enhancing Wnt signaling by stabilizing *β*-catenin.^18^ These CBC/Lgr5+ ISCs reside primarily at positions +1 to +3 above the crypt base, where they support self-renewal and generate epithelial progenitors that enter the transit-amplifying (TA) compartment.^19^ Upon division, these progenitors undergo rapid proliferation within the TA compartment, followed by upward migration along the crypt-villus axis and progressive differentiation into mature epithelial lineages.^5^ Of note, Paneth cells, which flank ISCs, secrete antimicrobial peptides, such as *α*-defensins and lysozyme, and produce essential niche factors, including the Wnt activator Wnt3, transforming growth factor-*α* (TGF-*α*), and Notch ligands that are critical for ISC proliferation.^20^ On the other hand, a population of dormant or quiescent stem cells, referred to as ‘+4’ cells due to their location at the fourth position above the crypt base, expresses multiple markers, including Bmi1, Hopx, and Lrig1. These cells remain inactive under homeostatic conditions but can be activated in response to tissue injury or environmental stress to facilitate tissue regeneration and repopulate the Lgr5+ ISC pool.^21-23^ Interestingly, interconversion between these stem cell populations has been demonstrated, with +4 cells capable of converting into CBC cells and vice versa, indicating a remarkable degree of functional plasticity.^24^ ^,^ ^25^ Recent studies using advanced methodologies, such as high-resolution single-cell profiling and *in vivo* lineage tracing, have begun to call into question the long-held belief that Lgr5+ CBC cells are the only ISC population.^26-29^ For instance, following the targeted ablation of CBC cells by diphtheria toxin administration in mice that express the human diphtheria toxin receptor gene under control of Lgr5 regulatory regions, Lgr5-negative progenitor populations were capable of driving intestinal regeneration.^27^ Complementing this, Malagola et al. showed that Lgr5 expression is not indispensable for ISC identity or function, and that stem cell potential extends beyond the crypt base to include the isthmus region.^26^ Furthermore, a recent report by Kirino et al. described bidirectional interconversion between CBCs and so-called revival stem cells (revSCs). They showed that upon injury, absorptive enterocytes can reprogram into revSCs, a highly stress-tolerant stem cell population, and regenerate CBCs.^30^ These findings support an alternative model of intestinal epithelial organization in which ISC potential is distributed more broadly than previously thought, encompassing additional Lgr5-negative populations that contribute to epithelial maintenance and repair under inflammatory conditions.

**Figure 1.**
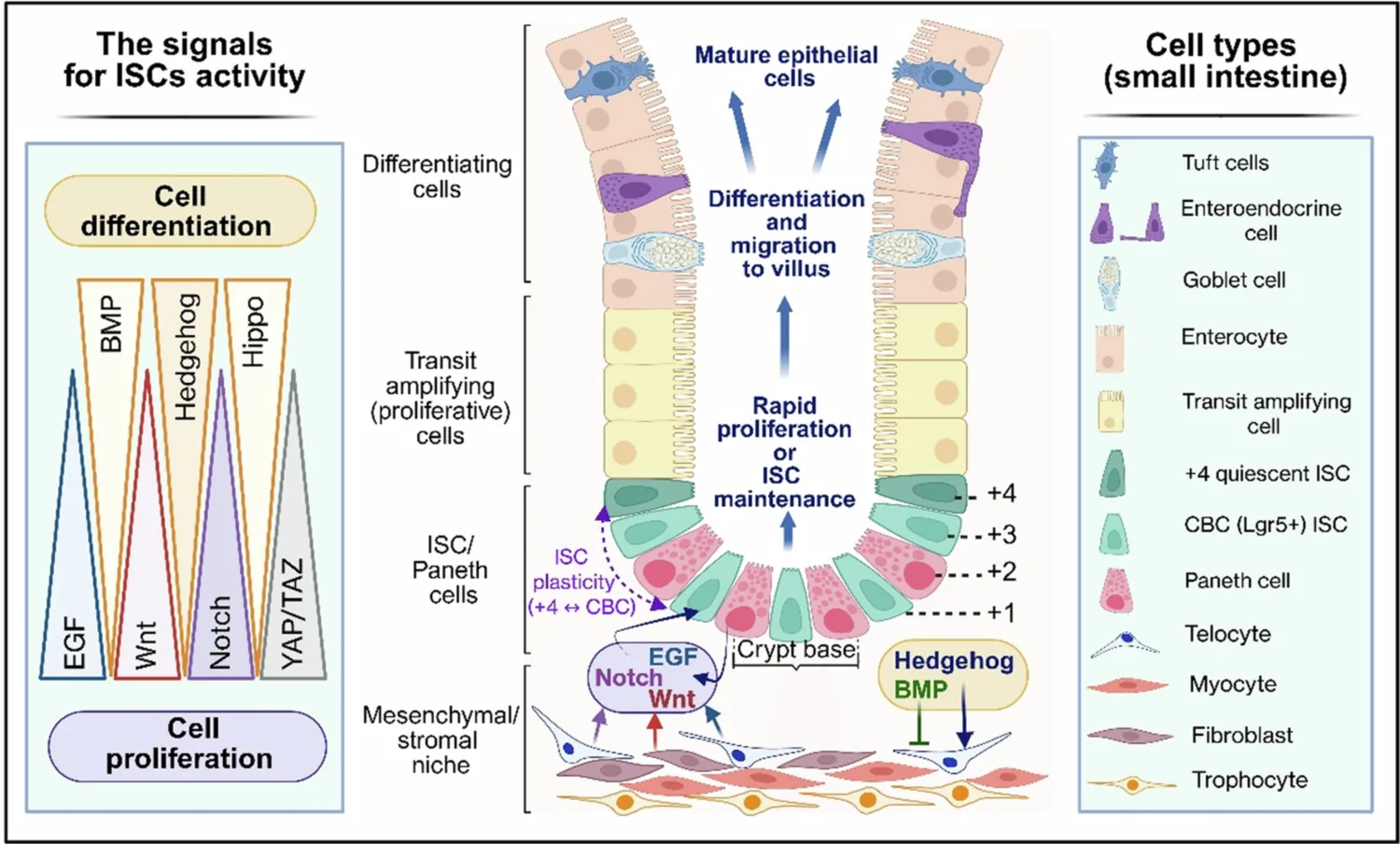
Organization of the small-intestinal crypt-villus axis and signaling pathways regulating ISC activity. Schematic representation of the small-intestinal crypt–villus axis illustrating the spatial organization of ISCs, proliferative progenitors, differentiated epithelial cells, and the surrounding mesenchymal/stromal niche. CBC/Lgr5+ ISCs are located predominantly at positions +1 to +3 above the crypt base, interspersed with Paneth cells, while the +4 population represents a quiescent ISC/reserve-like compartment. The figure depicts ISC maintenance and self-renewal at the crypt base, followed by rapid proliferation of progenitors within the TA compartment, upward migration along the crypt–villus axis, and progressive differentiation into mature epithelial lineages, including enterocytes, goblet cells, enteroendocrine cells, and tuft cells. The bidirectional arrow between +4 and CBC populations illustrates ISC plasticity and interconversion. The mesenchymal/stromal niche, comprising telocytes, myocytes, fibroblasts, and trophocytes, provides additional signals that contribute to ISC maintenance and epithelial differentiation. Key signaling pathways involved in ISC regulation are depicted, including EGF, Wnt/β-catenin, Notch, BMP, Hedgehog, and Hippo/YAP-TAZ signaling, highlighting their roles in epithelial proliferation, ISC maintenance, and differentiation. The left panel summarizes the principal signaling inputs associated with ISC proliferation and differentiation, whereas the right panel identifies the major epithelial and niche cell populations of the small intestine. Figure created with BioRender. Abbreviations: ISC, intestinal stem cell; CBC, crypt base columnar; TA, transit-amplifying; EGF, epidermal growth factor; Wnt, Wingless-related integration site; BMP, bone morphogenetic protein; YAP, Yes-associated protein; TAZ, transcriptional co-activator with PDZ-binding motif.

## Intrinsic signals shaping ISC function

ISC fate and activity are controlled by several conserved signaling pathways, including wingless-related integration site (Wnt)/β-catenin, Notch, bone morphogenetic protein (BMP), Hedgehog, epidermal growth factor (EGF), Hippo signaling pathway (Hippo)/yes-associated protein (YAP), and Nuclear factor kappa-light-chain-enhancer of activated B cells (NF-κB).^31^ The principal signaling pathways that regulate ISC maintenance, proliferation, and differentiation within the crypt-villus niche are depicted in Figure 1. Among these pathways, the canonical Wnt/β-catenin pathway plays a central role in regulating ISC function and maintaining the balance among self-renewal, proliferation, and differentiation.^32^ ^,^ ^10^ Wnt signaling inhibits *β*-catenin degradation, resulting in its accumulation and subsequent translocation to the nucleus, where *β*-catenin associates with T-cell factor/lymphoid enhancer-binding factor (TCF/LEF) transcription factors to activate Wnt-responsive genes involved in ISC maintenance and intestinal epithelial homeostasis.^33^ ^,^ ^32^ ^,^ ^34^ In addition, Wnt/β-catenin signaling contributes to the maturation of Paneth cells, which, in turn, produce Wnt ligands, such as Wnt3, and growth factors, such as EGF and Notch ligands, which support a local signaling milieu that is critical for ISC maintenance.^35^ ^,^ ^36^ Thus, the Wnt/β-catenin pathway is essential not only for maintaining the self-renewal capacity of ISCs but also plays an important role in the maturation of Paneth cells at the crypt base.

Notch signaling is another important regulator of ISC maintenance, proliferation, and lineage specification. It is mediated by single-pass transmembrane receptors and Delta/Serrate/Lag2 (DSL) family ligands such as Dll1, Dll3, Dll4, Jagged1, and Jagged2, preserves ISC stemness and directs differentiation toward absorptive enterocyte lineages.^31^ Canonical Notch signaling is initiated when membrane-bound ligands such as DLL1 and DLL4 bind Notch receptors on neighboring epithelial cells, triggering receptor cleavage and release of the Notch intracellular domain (NICD). NICD then translocates to the nucleus, where it associates with the transcriptional regulator RBPJ/CSL to activate target genes, including *HES1*.^37^ ^,^ ^38^ In the intestinal epithelium, Notch activity maintains the proliferative ISC/progenitor compartment and regulates the balance between absorptive and secretory differentiation. Notch-dependent *HES1* expression suppresses *ATOH1*, thereby favoring absorptive enterocyte differentiation, whereas reduced Notch activity promotes secretory cell differentiation, including goblet, Paneth, and enteroendocrine lineages.^38^ Notch also interacts with Wnt/β-catenin signaling to coordinate ISC maintenance and epithelial differentiation.^5^ Thus, alterations in microbial metabolites or inflammatory signals associated with dysbiosis may influence ISC homeostasis by modulating Notch activity and its interaction with other regulatory pathways.

The BMP and Hedgehog pathways act as negative regulators, restricting ISC proliferation and facilitating differentiation. BMP, a member of the transforming growth factor-*β* (TGF-*β*) superfamily, signals through the dimerization of BMP type I and type II receptors (BMPR1 and BMPRII), leading to the phosphorylation and subsequent activation of receptor-regulated SMADs (R-SMADs)^39^. R-SMADs then form heterotrimeric complexes with the common mediator SMAD, SMAD4 (co-SMAD), and translocate to the nucleus, where they regulate the expression of target genes. BMP signaling has been implicated in maintaining intestinal homeostasis and restraining intestinal tumorigenesis^39^. On the other hand, Hedgehog signaling primarily acts through a paracrine mechanism in the intestine, whereby Hedgehog ligands produced by intestinal epithelial cells signal to mesenchymal cells. This pathway regulates mesenchymal cell proliferation and controls the production of WNT and BMP ligands, thereby establishing an epithelial-mesenchymal feedback loop that maintains intestinal homeostasis and promotes epithelial differentiation.^40^ ^41^

EGF and Hippo/YAP signaling pathways govern epithelial cell growth, survival, and regenerative potential. The EGF ligand family comprises multiple growth factors, including TGF-*α*, AREG, EREG, that signal through four ErbB receptors (EGFR/ErbB1, ErbB2, ErbB3, and ErbB4).^41^ In the intestine, mesenchymal cells produce NRG1, NRG2, AREG, and EREG, which act paracrinely on intestinal epithelial cells, whereas epithelial cells produce EGF, BTC, HB-EGF, and TGF-*α* to regulate epithelial functions in an autocrine manner.^42^ EGF–ErbB signaling activates downstream mitogen-activated protein kinase (MAPK), PI3K–AKT, and JAK–STAT pathways, thereby promoting ISC proliferation, epithelial regeneration, and maintenance of intestinal homeostasis.^41^ ^,^ ^43^ On the other hand, the Hippo/YAP pathway is a key regulator of intestinal epithelial homeostasis, stem cell activity, and tissue regeneration. Activation of the core Hippo kinases MST1/2 and LATS1/2 promotes phosphorylation and cytoplasmic retention of the transcriptional co-activators YAP and transcriptional co-activator with PDZ-binding motif (TAZ), thereby limiting their transcriptional activity. In contrast, reduced Hippo activity allows YAP/TAZ to translocate to the nucleus, where they interact with transcriptional enhanced associate domain (TEAD) transcription factors to promote genes involved in stem cell proliferation, survival, and regeneration. In the intestine, YAP/TAZ activity supports ISC and progenitor cell expansion and is particularly important for epithelial repair following injury; however, persistent YAP/TAZ activation can promote excessive tissue growth and tumorigenesis.^44^ ^,^ ^45^

NF-κB integrates immune and epithelial signals, particularly in response to stress or inflammatory stimuli, thereby linking environmental cues to ISC function.^46^ Following exposure to microbial components or inflammatory cytokines, the IKK complex facilitates IκB degradation, releasing NF-κB transcription factors to enter the nucleus and modulate genes associated with inflammation, cell survival, proliferation, and epithelial regeneration.^47^ Within the intestinal epithelium, controlled NF-κB signaling contributes to maintenance of barrier function and stem cell activity, whereas prolonged or dysregulated activation may enhance inflammatory responses and abnormal epithelial proliferation.^48^,​​​​​​^47^

## Microbial metabolite-mediated ISC regulation

Advances in metabolomics, particularly through the application of mass spectrometry and nuclear magnetic resonance, have expanded our understanding about the metabolic output of the gut microbiota.^49^ This output encompasses a vast repertoire of bioactive compounds, including SCFAs, indoles, lactate, and secondary bile acids produced via microbial fermentation of dietary- and host-derived substrates.^33^ ^,^ ^50^ According to the Human Microbial Metabolome Database (MiMeDB) (https://mimedb.org), microbial communities generate up to 15,000 distinct small molecules, either autonomously or via co-metabolism with the host.^51^ Growing evidence suggests that gut metabolites play a significant role in the regulation of ISCs, thereby modulating proliferation, differentiation, and the overall epithelial renewal.^8-10^ ^,^ ^15^ ^,^ ^52^ ^,^ ^11^ ^,^ ^53-55^ These metabolites can have stimulatory, inhibitory, or both effects on ISCs through altering various signaling pathways (Table 1 and Figure 2).

**Figure 2.**
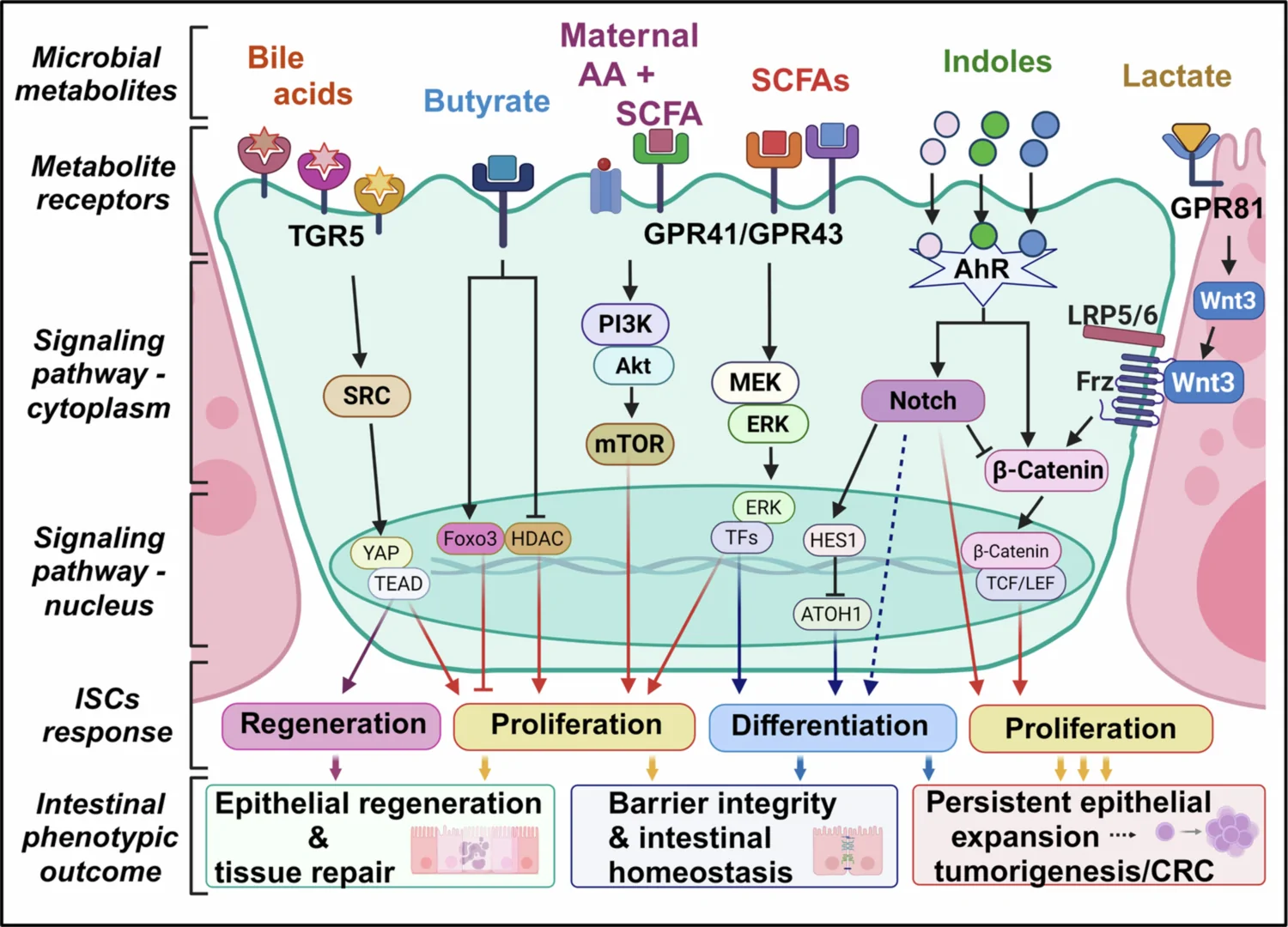
Microbiota-derived metabolites regulate ISC signaling and downstream intestinal phenotypes. Bile acids, butyrate, maternal AAs plus SCFAs, SCFAs, indoles, and lactate interact with their respective epithelial receptors or intracellular targets to engage distinct signaling pathways. Bile acids activate TGR5 and downstream SRC-YAP/TEAD signaling, promoting ISC regeneration. Butyrate modulates nuclear Foxo3 and HDAC activity, thereby influencing ISC proliferation. Maternal AAs and SCFAs activate the PI3K-AKT-mTOR pathway, whereas SCFAs signal through GPR41/GPR43-MEK-ERK and associated transcription factors to regulate ISC proliferation and differentiation. Microbiota-derived indoles activate AhR and subsequently modulate Notch and Wnt/β-catenin signaling, thereby influencing ISC differentiation and proliferation. Lactate signals through GPR81 and Wnt3/β-catenin to promote ISC proliferation. These metabolite-responsive pathways converge on distinct ISC phenotypes, including regeneration, proliferation, and differentiation, which contribute to epithelial regeneration and tissue repair and maintenance of intestinal barrier integrity and homeostasis. Persistent or dysregulated activation of ISC-associated signaling, particularly pathways that sustain epithelial proliferation, may lead to persistent epithelial expansion and contribute to tumorigenesis and CRC. Figure created with BioRender. Abbreviations**:** AhR, aryl hydrocarbon receptor; AKT, protein kinase B; AA, amino acid; CRC, colorectal cancer; ERK, extracellular signal-regulated kinase; Foxo3, forkhead box O3; GPR41/43, G-protein-coupled receptor 41/43; GPR81, G-protein-coupled receptor 81; HDAC, histone deacetylase; ISC, intestinal stem cell; MEK, mitogen-activated protein kinase kinase; mTOR, mechanistic target of rapamycin; PI3K, phosphoinositide 3-kinase; SCFAs, short-chain fatty acids; SRC, proto-oncogene tyrosine-protein kinase Src; TEAD, Transcriptional Enhanced Associate Domain; TCF/LEF, T-cell factor/lymphoid enhancer factor; TGR5, Takeda G protein-coupled receptor 5; Wnt, wingless-related integration site; YAP, yes-associated protein.

Microbiota-derived metabolites reaching the intestinal epithelium arise through several interconnected metabolic routes. Some are generated from dietary substrates, such as SCFAs from carbohydrate fermentation and indole derivatives from microbial tryptophan metabolism. A second group comprises microbial metabolic intermediates, such as lactate, which can be further transformed through interspecies metabolic cross-feeding. Others originate from host-derived molecules that are subsequently modified by microbes, as exemplified by the conversion of primary bile acids into secondary bile acids.^56-58^ These pathways are interconnected, and metabolite availability at the epithelial surface depends on substrate availability, microbial community composition, metabolic interactions, intestinal location, and utilization by host cells. Thus, considering both the metabolic origin and local availability of microbial metabolites provides a useful framework for understanding their diverse effects on ISC proliferation, differentiation, and epithelial renewal. For clarity, we have organized the metabolites discussed in this review into three broad groups according to their predominant metabolic origin:

### Microbial conversion of dietary substrates

**SCFAs:** SCFAs are fatty acids with fewer than six carbon atoms that contain a carboxyl group and a hydrocarbon chain. They are primarily produced in the large intestine by anaerobic bacterial fermentation of complex carbohydrates, such as fibers, that remain undigested in the small intestine.^59^ The most common of these are acetate, propionate, and butyrate, which account for 90% of SCFAs produced by gut microbiota and provide instant energy to host cells due to their rapid absorption and metabolism.^60^ ^,^ ^61^ Evidence from germ-free and antibiotic-treated mouse models showed that reduced SCFA levels in the cecal and colonic contents are associated with impaired proliferative and migratory activity of intestinal epithelial cells (IECs) in intestinal crypts.^53^ Interestingly, while these conditions did not markedly affect the numbers of ISCs or goblet and Paneth cell populations, they significantly decreased Bromodeoxyuridine (BrdU)+ proliferative cells and migration of IECs along the crypt–villus axis, suggesting that SCFAs primarily promote the proliferation of progenitor cells and IEC turnover rather than terminal differentiation.^53^ Moreover, SCFA supplementation in the drinking water restored IEC proliferation and migration in antibiotic-treated mice. Complementary *in vitro* studies with intestinal organoids further demonstrated that individual SCFAs (acetate, propionate, or butyrate) enhanced organoid growth, with the combined SCFA cocktail exerting the most pronounced effect.^53^ Given that SCFAs act as ligands for G-protein-coupled receptors (GPRs), including GPR41 and GPR43, in a dose-dependent manner,^62-64^ pharmacological activation of these receptors using the GPR41 agonist CPC or the GPR43 agonist 4-CMTB promoted mouse intestinal organoid development.^53^ Similarly, administration of *Akkermansia muciniphila,* a mucin-degrading commensal bacterium that produces acetate and propionate, resulted in accelerated proliferation of Lgr5+ ISCs in mice. The cecal contents from *A. muciniphila*-treated mice enhanced small intestinal organoid formation, which was abolished by treatment with GPR41/43 antagonists.^65^ Overall, these data indicate a direct and potentially additive or synergistic role for SCFAs in promoting epithelial renewal and ISC activity. In line with these findings, Dang et al. showed in mice that maternal gut microbiota composition and its associated metabolites during gestation can influence ISC populations in the offspring, leading to lasting functional effects.^8^ Exposure of human mothers to *A. muciniphila*, increased serum levels of SCFAs, including acetic, propionic, and butyric acid, as well as amino acids (glycine, glutamine, arginine).^8^ More importantly, when intestinal organoids derived from Lgr5-2A-CreERT2-R26-tdTomato mice, a non-variegated reporter-based *in vivo* lineage-tracing model, were treated with serum from *A. muciniphila*-exposed versus control mouse dams, a higher proportion of Tomato-positive ISCs was observed in the former group, implying that maternal microbial-derived metabolites, especially SCFAs, contribute to the intergenerational programming of offspring ISCs.^8^ Considering the crucial role of the gut microbiota in shaping the host metabolite pool, the elevated SCFA levels in *A. muciniphila*-exposed mothers likely reflect increased activity of SCFA-producing taxa, such as Firmicutes.^66^ Mechanistically, elevated levels of maternal SCFAs and amino acids imprinted distinct transcriptomic profiles on the mechanistic target of rapamycin (mTOR) pathway in offspring ISCs. Pharmacological inhibition of mTOR during pregnancy reversed these microbiota-driven effects, emphasizing the role of maternal microbial signaling in ISCs function and revealing a potentially modifiable axis for early-life interventions.^8^

Gut microbiota-derived propionate/propionic acid has been associated with regulating the stemness of ISCs and restoring the expression of ISC markers.^67^ ^,^ ^68^ Administration of propionate through drinking water in mice markedly diminished dextran sulfate sodium (DSS)-associated tissue damage while enhancing crypt proliferation, as seen by an elevation in cells expressing Ki-67, a marker of tissue regeneration.^67^ Furthermore, treating the intestinal organoids with propionate counteracted the DSS-induced decreased expression of the ISC markers, such as Olfm4 and Lgr5 in organoids.^67^ Likewise, exposure to propionate or propionic acid promoted the development of organoids, resulting in an increased number of Lgr5+ ISCs.^68^ 5-ethynyl-2’-deoxyuridine (EdU) tests and Ki-67 labeling further demonstrated that these treatment regimens stimulated crypt cell proliferation in organoids.^68^ These results together infer that propionate and/or propionic acid play significant roles in ISC-mediated IEC homeostasis and epithelial development.

It is worth noting that SCFAs can also inhibit the proliferation of ISCs and their niche cells.^9^ Kaiko et al. performed a multistep blind screening of a library of microbial metabolites produced or induced by the murine intestinal microbiota to evaluate their effects on ISC activity.^9^ Among the metabolites identified, butyrate, a major SCFA generated by bacterial fermentation of dietary fiber abundant in the colon,^69^ emerged as a potent inhibitor of intestinal stem/progenitor-cell proliferation at physiologically relevant concentrations. This effect was demonstrated *in vitro* using primary colonic epithelial cells isolated from Cdc25A-click beetle red reporter mice and cultured under conditions favoring the expansion of dividing Lgr5+ ISCs.^9^ Moreover, *in vivo* exposure of ISCs/progenitor cells to butyrate, through inducing colon injury with DSS in mice or by applying exogenous butyrate to zebrafish, which naturally lack colonic crypts and the microbiota-derived enzymes required to produce butyrate, resulted in marked inhibition of cell proliferation and impaired wound healing.^9^ Mechanistically, butyrate-mediated inhibitory action, however, was reversed by inhibiting the activities of the transcription factor Foxo, which regulates cell cycle genes. Deletion of Foxo3, but not Foxo1, in colonic stem/progenitor cells resulted in abolition of butyrate inhibitory effects. In line with these findings, delivering Foxo inhibitor enemas to mice with DSS-induced mucosal damage abolished the inhibitory effect of butyrate on epithelial proliferation.^9^ These data suggest that butyrate can act as a potent inhibitory regulator of ISC function through a Foxo3-dependent mechanism.

Butyrate may have additional effects that distinguish it from other major SCFAs. It is a relatively potent endogenous histone deacetylase (HDAC) inhibitor compared with other major SCFAs, providing a potential mechanism for altering transcriptional programs that regulate epithelial proliferation and differentiation.^70^ In addition, its oxidation by colonocytes consumes oxygen and contributes to the physiologically hypoxic epithelial environment, thereby activating hypoxia-inducible factor (HIF)-dependent pathways.^71^ Thus, butyrate may influence ISC function through both epigenetic regulation and modulation of the metabolic and oxygen landscape of the epithelial niche, although whether epithelial hypoxia directly contributes to ISC inhibition remains unclear. Nevertheless, the effects of butyrate on intestinal epithelial proliferation appear to be context- and dose-dependent, which may help reconcile apparently conflicting observations across experimental systems. Lower concentrations tend to promote epithelial metabolism and proliferation, whereas higher concentrations can exert suppressive effects.^72^ ^,^ ^73^ Such differences may reflect variation in butyrate concentration, cellular metabolic state, spatial accessibility within the intestinal compartment, microbial composition, and experimental model.

Collectively, these observations suggest that the proliferative or inhibitory effects of butyrate depend on its local availability and accessibility to the ISC niche, together with the metabolic state and differentiation of epithelial cells. Efficient oxidation of butyrate by differentiated colonocytes can limit its access to ISCs and support epithelial metabolism, whereas increased intracellular availability may enhance HDAC-dependent transcriptional effects and, in ISCs, promote Foxo3-associated cell-cycle suppression.^9^ ^,^ ^72^ Thus, rather than assuming a single universal concentration threshold, the proliferative-to-inhibitory response is likely determined by the local concentration and accessibility of butyrate in relation to the metabolic state and architecture of the ISC niche. The precise threshold and relative contribution of these mechanisms, however, remain incompletely defined.

**INDOLES**: Indoles are derivatives of indole whose core is made up of a pyrrole ring fused to a benzene ring, and they have long piqued the interest of scientists due to their enormous biologically active role in various physiological processes.^74^ ^,^ ^75^ They play crucial roles as signaling molecules and metabolic intermediates, influencing mood, sleep, circadian rhythms, gut barrier function, immune regulation, and neuronal and endocrine processes.^74^ ^,^ ^75^ Furthermore, tryptophan, an essential amino acid and the principal precursor of indole, is predominantly absorbed in the small intestine when ingested with dietary sources. Gut microbiota catabolizes approximately 4-6% of total ingested tryptophan into indoles, such as indole-3-acetic acid (I3A) and indole-3-aldehyde (I3Ald)^76^ ^,^ ^77^ and these indoles have been found to have a dual role, both stimulatory and detrimental, in maintaining intestinal homeostasis.^78^

A major mechanism by which indoles influence intestinal epithelial biology is through the aryl hydrocarbon receptor (AhR), a ligand-activated transcription factor expressed in intestinal epithelial and immune cells (Table 1, Figure 2). AhR responds to diverse microbial and dietary indole derivatives and, upon ligand binding, regulates genes involved in epithelial barrier integrity, immune homeostasis, and cellular differentiation.^79^ ^52^ ^,^ ^76^ In ISC biology, AhR signaling is ligand- and context-dependent and can interact with key pathways such as Wnt/β-catenin and Notch. For example, indole-3-carbinol promotes goblet-cell differentiation through AhR-dependent modulation of Wnt and Notch signaling, while I3Ald activates AhR and improves epithelial barrier function and regeneration in experimental colitis.^80^ ^,^ ^81^ Thus, AhR represents an important link between microbial tryptophan metabolism and intestinal epithelial homeostasis, although the effects of individual indole metabolites on ISCs vary according to their specific molecular and cellular contexts.

Recent research has shown that I3A has the ability to significantly inhibit ISC proliferation and turnover.^11^ ^,^ ^16^ Zhang et al. investigated the biological effects of I3A packaged within outer membrane vesicles (OMVs) derived from *Acinetobacter radioresistens*, a dominant taxon residing in the intestinal crypt niche. Using both small and large intestinal organoids, they demonstrated that OMVs from *A. radioresistens* significantly reduced epithelial proliferation, as indicated by decreased numbers of EdU+ cells.^16^ Importantly, OMVs derived from *A. radioresistens* ΔtrpC mutant strain deficient in I3A production failed to suppress organoid proliferation, confirming that I3A is essential for the antiproliferative activity of these vesicles. This I3A-dependent suppression was also observed in human intestinal organoids, suggesting a conserved effect across species.^16^ Furthermore, OMVs from *A. radioresistens* or I3A downregulated the expression of Wnt target genes, such as c-Myc, Cyclin D1, and Ascl2. Using Axin2-2A-EGFP reporter mice, it was further shown that *A. radioresistens* OMVs decreased the proportion of Axin2+ ISCs compared to controls or OMVs from a ΔtrpC mutant strain deficient in I3A production. This implicated I3A as a specific microbial suppressor of Wnt signaling.^16^

Since I3A is also an AhR ligand, AhR-dependent transcriptional responses may represent an additional mechanism linking microbial tryptophan metabolism to epithelial signaling. However, the relative contribution of AhR versus direct modulation of Wnt/β-catenin signaling to the ISC phenotype requires further investigation. An interesting study by Wei et al. used a chronic restraint stress (CRS) mouse model to show that psychological stress induces I3A production by *Lactobacillus murinus*, which impairs ISC lineage commitment through ISC-intrinsic mechanisms.^11^ In addition, elevated fecal I3A levels were observed in patients experiencing mental distress, annexing stress-induced microbial metabolism to altered ISC dynamics in humans. This suggests a mechanistic pathway by which stress influences gut homeostasis via microbial production of I3A, positioning the brain-gut-microbiota axis as a promising target for therapeutic intervention in stress-related gastrointestinal disorders.^11^ To elucidate the mechanistic pathway through which I3A exerts its effects on ISCs, single-cell RNA sequencing (scRNA-seq) was conducted on intestinal organoids treated with I3A. Multiple analyses, including Pseudotime, DEGs, and KEGG pathway enrichment, revealed a decreased commitment to the secretory lineage among ISCs, dysregulation of genes involved in mitochondrial integrity (Ndufab1, mt-Atp8, mt-Rnr1/2), and impaired oxidative phosphorylation (OXPHOS) capacity, consistent with observed disruptions in mitochondrial morphology.^11^ Similar to I3A, cell proliferation assays revealed that another indole, indole-3-carbinol, suppressed intestinal organoid formation, most likely by inhibiting ISC proliferation.^80^

In contrast, I3Ald has been reported to promote ISC differentiation and proliferation in both piglet and mouse models.^81^ ^,^ ^82^ Piglets with diarrhea had reduced microbial synthesis of tryptophan metabolites in their feces compared to healthy piglets, and dietary supplementation with I3Ald ameliorated intestinal barrier integrity in diarrheal piglets.^82^ I3Ald is also implicated in improving intestinal epithelial barrier in a mouse model of DSS-induced colitis.^81^ In a porcine intestinal organoid system, exposure to I3Ald significantly increased both budding efficiency and organoid surface area, accompanied by upregulation of Lgr5, indicating that I3Ald plays a promoting role in ISC expansion.^82^ The ISC-promoting effects of I3Ald differ from the inhibitory effects of other indoles, such as I3A, as previously discussed, and could be attributed to a variety of factors, including experimental design, dosage or experimental models used.

Recently, a new role for indole-3-propionic acid (IPA) and indole-3-lactic acid (ILA) in ISC-mediated epithelial repair has been described.^83^ ^,^ ^84^ The IPA/ILA-β‑hydroxybutyrate (BHB)-ISC axis is a newly discovered gut microbiota–metabolite–stem cell regulatory pathway essential for regeneration of intestinal epithelium. In an elegant study, Zhang et al. showed that *Blautia coccoides*-derived ILA can be converted through cross-feeding into IPA by commensals such as *Peptostreptococcus russellii* or *Clostridium sporogenes*, leading to epithelial BHB production, activation of HOPX+ reserve ISCs, restoration of the Lgr5+ ISC pool, and improved mucosal healing in a mouse model of DSS-induced colitis.^83^ Another study by the same group showed that IPA activates PPARα in IECs, induces HMGCS2 expression, increases BHB production, and thereby promotes Lgr5+ ISC-dependent regeneration. Restoration with either IPA or BHB attenuated inflammation and barrier defects in mouse models of chemically induced colitis, suggesting that IPA levels can be therapeutically targeted in inflammatory bowel disease and other barrier disorders.^84^

### Microbial metabolic products and cross-feeding

**LACTATE:** Lactate represents an important intermediary metabolite within the gut microbial metabolic network. It is the conjugate base of lactic acid and predominantly produced by a group of bacterial species known as lactic acid bacteria (LAB), which ferment simple carbohydrates such as glucose and sucrose as a substrate.^85-87^ It can subsequently be consumed or transformed by other microbial species. Consequently, intestinal lactate levels reflect both its microbial production and its utilization by the surrounding microbial community. Beyond its role as a metabolic intermediate, lactate can act as a signaling molecule that influences intestinal epithelial cells and ISCs through pathways including GPR81 and Wnt/β-catenin signaling.^7^ ^,^ ^15^ ^,^ ^88^

Okada et al. previously demonstrated that lactate is important for the proliferation of colonic epithelial cells.^88^ Wu et al. used porcine jejunal epithelial organoids to show that treating organoids with lactate for 48 hours significantly increased the growth and proliferation of intestinal organoids compared with mock-treated controls in a dose-dependent fashion.^89^ Mechanistically, lactate activated Wnt/β-catenin signaling in a GPR81-dependent manner to promote ISC-mediated epithelial proliferation.^7^ A recent study by Lee et al. performed in-depth research on how lactate affects ISCs function under steady-state and disease conditions using mouse models and organoids.^15^ Oral administration of LAB, compared with PBS-fed controls, in Lgr5-GFP mice resulted in increased crypt heights and higher numbers of Lgr5+ ISCs, Paneth cells (Lyz1+), goblet cells (Muc2+), Ki-67+ proliferating cells, and Olfm4+ cells in the small intestine, followed by significantly enhanced levels of lactate in both the small and large intestine. Furthermore, small intestinal crypts cultured with probiotic culture supernatant or lactate produced larger organoids and higher Lgr5+ ISC counts, significantly increased expression of Wnt/β-catenin-related genes including Wnt3, Ctnnb1, Axin2, and GSK3β. Of note, the effect of lactate on the proliferation of ISCs was significantly greater in newborn mouse small intestine organoids compared to those derived from adult mice. To further confirm the role of lactate, adding BHB inhibited organoid growth in lactate-supplemented conditions.^15^ Of note, metabolic lactate from Paneth cells has been shown to support Lgr5+ ISC function by maintaining enhanced mitochondrial oxidative phosphorylation.^90^ Additionally, administering lactate protected mice from significant intestinal damage caused by radiation and chemotherapy, thereby steering the survival of Lgr5+ ISCs.^15^ Thus, lactate plays a crucial role in regulation of ISC function that may have important clinical implications for developing prophylactic and therapeutic strategies to reduce gut injury during therapeutic regimens.

However, the effects of lactate on intestinal homeostasis are context-dependent and may not always be beneficial. Physiological lactate concentrations can support epithelial regeneration and modulate immune responses, whereas sustained lactate accumulation in hypoxic or inflamed tissues is frequently associated with extracellular acidification and altered inflammatory signaling. Lactate can influence pathways involving GPR81, NF-κB, HIF-1α, and inflammasome activity, with its effects varying according to concentration, cellular context, metabolic state, and duration of exposure.^91-93^ Indeed, lactate can exert context-dependent immunomodulatory effects, with studies reporting both anti- and pro-inflammatory activities depending on cell type, receptor/transport mechanism, metabolic state and experimental setting.^92^ ^,^ ^94^ Thus, while microbiota-derived lactate may promote ISC expansion and epithelial repair under physiological conditions, excessive or sustained lactate accumulation in an acidic and inflammatory microenvironment may produce opposing effects. This context dependence should be considered when interpreting the contribution of lactate to intestinal epithelial regeneration and repair.

### Microbial modification of host-derived molecules

**BILE ACIDS:** Primary bile acids are produced in the liver and serve as emulsifiers that aid in the digestion of fats in the small intestine. Although the bulk of bile acids are reabsorbed in the small intestine and recirculated to the liver via enterohepatic circulation, a small percentage travels to the colon.^95^ The action of microbiota on the bile acids in the colon gives rise to secondary bile acids. In humans these comprise mainly deoxycholic acid (DCA) and lithocholic acid (LCA).^95^ Thus, the intestinal microbiota contributes to the composition and biological activity of the bile acid pool rather than being the primary source of bile acid synthesis.

Sorrentino et al. conducted extensive research into the role of bile acids in ISC regulation in mice and organoid cultures.^54^ Mice fed high-fat diet (HFD) for 21 days showed increased expression of the ISC marker Lgr5 in duodenal samples, followed by the accumulation of bile acids in feces. Furthermore, freshly isolated mouse intestinal crypts were exposed to a crude bile extract or secondary bile acids (DCA or LCA), for 6 days, which significantly promoted *de novo* crypt growth, as evidenced by an increase in the number of buds per organoid. On the other hand, high doses of LCA resulted in a significant drop in organoid counts.^54^ Thus, the actions of bile acids on ISC activities seem to be dose-dependent. Additionally, the bile acid-responsive membrane receptor, Takeda G protein-coupled receptor 5 (TGR5), also referred to as GPBAR1, is expressed throughout the intestinal tract and regulates various physiological functions, including intestinal motility and immunity.^96^ TGR5^ISC^^−/−^ mice, with disrupted TGR5 in Lgr5+ ISCs had severe DSS-induced colitis compared to mice without TGR5 disruption in ISCs, whereas ISCs treated with the TGR5 agonist INT-777 showed enhanced activation of YAP1 and its upstream regulator proto-oncogene tyrosine-protein kinase Src (SRC). Furthermore, inhibitors of YAP1 and SRC inhibited organoid development caused by TGR5 activation.^54^ Collectively, bile acids illustrate how microbial metabolism can reshape a host-derived signaling pool and thereby alter ISC behavior. The microbial contribution is therefore not simply the production of a metabolite but the biotransformation of a host-derived molecule into a chemically and biologically distinct set of signaling molecules. Through receptors such as TGR5 and downstream pathways including SRC and YAP, these microbiota-modified bile acids provide a direct route by which luminal metabolic information can influence ISC renewal and epithelial repair.

Microbial effects on ISCs are highly context-dependent and vary with metabolite concentration, intestinal region, epithelial injury, inflammation, age, and diet. As an example, butyrate can support epithelial metabolism under physiological conditions but suppress ISC/progenitor proliferation when it gains greater access to the crypt compartment, whereas secondary bile acids can promote regeneration through TGR5 signaling at appropriate concentrations but impair epithelial growth at excessive levels.^9^ ^,^ ^54^ ISC responsiveness may also vary with developmental stage, as lactate produces a greater proliferative response in neonatal than adult intestinal organoids.^15^ Diet further influences these responses by altering microbial substrate availability and metabolite production. Thus, a signal that promotes repair after acute injury may have limited or adverse effects during homeostasis or chronic inflammation, particularly in tumor-prone settings. These findings highlight the importance of dose, spatial distribution, timing, and host context when evaluating microbiota-derived signals as regulators or therapeutic targets of ISC function.

**Table 1.** Impact of microbial metabolites on ISC function and the molecular pathways by which metabolites exert their actions.

Membrane receptor signaling					
Metabolite	Receptor	Effect on ISC	Experimental model	Mechanism/evidence	References
**Acetate, propionate**	GPR43 GPR41	↑/context-dependent	Mouse intestinal organoids; *in vivo* mouse intestine	Activation of GPR41 and GPR43. Evidence for effects on intestinal epithelial turnover exists, but direct ISC-autonomous regulation is less firmly established.	[58]
**Butyrate**	GPR41	↑/↓ context-dependent, dose-dependent	Mouse intestinal organoids; *in vivo* models	GPR41 mediates butyrate signaling in intestinal and immune cells; effects on ISC proliferation appear predominantly indirect/context-dependent.	[58]
**Lactate**	GPR81	↑	GF and conventional mice; pigs; mouse intestinal organoids; Paneth-cell/ISC niche models	Microbiota-derived lactate activates GPR81 and stimulates Wnt3 production in the ISC niche, promoting ISC expansion.	[15,88,89]
**Bile acids**	TGR5	↑	Lgr5+ ISC-derived mouse organoids; intestinal injury models	TGR5 signaling activates SRC–YAP1, promoting ISC renewal and epithelial regeneration.	[54]
**Nuclear receptor signaling**					
**Indole-3-aldehyde**	AhR	↑	HFD-fed mice; *ex vivo* mouse intestinal organoids; weaned piglets; porcine small-intestinal organoids	Indole-3-aldehyde activates AhR and promotes ISC proliferation/expansion, with increased ISC-associated markers.	[81,82]
**Indole-3-carboxaldehyde**	AhR	↑/regenerative	Mouse intestinal organoids; intestinal injury/radiation models	AhR-dependent signaling promotes epithelial regeneration and Lgr5+ ISC responses following injury.	[97]
**Indole-3-acetic acid**	AhR	↓	Mouse intestinal organoids; crypt-resident bacteria	Microbial indole-3-acetic acid –AhR signaling can restrain ISC turnover through regulation of Wnt/β-catenin signaling.	[16]
**Indole-3-carbinol**	AhR	↓ proliferation/↑ differentiation	Mouse crypt-derived organoids; Lgr5+ stem/TA cells; mice	Indole-3-carbinol inhibits organoid development and proliferation of Lgr5+ stem/TA cells while promoting secretory cell differentiation through Wnt/Notch regulation.	[80]
**Dietary lipids**	PPARδ/PPARβ	↑	HFD-fed mice; Lgr5+ ISC organoids	PPARδ activation promotes ISC self-renewal and proliferation.	[98]
**Bile acids**	FXR	↓/homeostatic	Mouse intestinal organoids; FXR-deficient mice; *in vivo* intestinal models	FXR activation limits excessive Lgr5+ ISC proliferation and protects against bile-acid-associated epithelial damage.	[99]
**Bile acids**	FXR/PXR	Context-dependent	Intestinal epithelial models and mice	Secondary bile acids signal through FXR and PXR as well as TGR5, integrating metabolic and epithelial responses.	[54]
**Metabolic and epigenetic regulation**					
**Butyrate**	HDAC inhibition → Foxo3	↓	Mouse intestinal organoids; intestinal stem/progenitor cells; mice	Butyrate suppresses proliferation of intestinal stem/progenitor cells, partly through HDAC inhibition and Foxo3 activation.	[9]
**Acetic acid, propionic acid, and butyric acid plus glycine, glutamine, and arginine**	mTOR signaling	↑ ISC proliferation	Mouse intestinal organoids; mice	Metabolites from maternal microbiota during pregnancy program offspring stem cells through mTOR pathway	[8]
**Pyruvate**	Mitochondrial pyruvate metabolism/MPC1	↓ mitochondrial oxidation → ↑ ISC self-renewal	Lgr5+ mouse ISCs; Lgr5-derived intestinal organoids; mice	MPC1 is low in Lgr5+ ISCs and increases during differentiation. MPC1 deletion increases the ISC pool and maintains stemness.	[100]
***β*-hydroxybutyrate**	HDAC inhibition/ketogenesis	↑ self-renewal/↓ differentiation toward Paneth cells	Mouse intestinal organoids; intestinal injury models; ketogenic diet	HMGCS2-derived *β*-hydroxybutyrate inhibits HDACs, reinforces Notch signaling and promotes ISC self-renewal at the expense of Paneth-cell differentiation.	[101]
**Ketone bodies**	HMGCS2-dependent ketogenesis	↑ ISC self-renewal	Mouse intestinal organoids; mice; high-fat/ketogenic diet models	Ketogenesis links nutrient availability to ISC self-renewal through *β*-hydroxybutyrate and epigenetic regulation.	[102]
**Indole-3-propionic acid**	PPARα → HMGCS2 → ketogenesis	↑	Mouse intestinal organoids; Lgr5+ ISCs; microbiota-colonized mice; intestinal injury models	Indole-3-propionic acid induces PPARα/HMGCS2-dependent ketogenesis, supporting ISC fitness and regeneration.	[84]
**Acetyl-CoA/cellular metabolism**	Histone acetylation	Context-dependent	Lgr5+ ISCs; intestinal organoids	Metabolite availability influences chromatin-modifying enzymes and histone acetylation, linking cellular metabolism to ISC state.	[103]

Abbreviations: AhR, aryl hydrocarbon receptor; Foxo3, for khead box O3; FXR, farnesoid X receptor; GF, germ-free; GPR41, G-protein-coupled receptor 41; GPR43, G-protein-coupled receptor 43; GPR81, G-protein-coupled receptor 81; HDAC, histone deacetylase; HFD, high-fat diet; HMGCS2, 3-hydroxy-3-methylglutaryl-CoA synthase 2; ISC, intestinal stem cell; Lgr5, leucine-rich repeat-containing G-protein-coupled receptor 5; mTOR, mechanistic target of rapamycin; MPC1, mitochondrial pyruvate carrier 1; PPAR, peroxisome proliferator-activated receptor; PXR, pregnane X receptor; SRC, proto-oncogene tyrosine-protein kinase Src; TA, transit-amplifying; TGR5, Takeda G-protein-coupled receptor 5; Wnt, Wingless-related integration site; Wnt3, Wnt family member 3; YAP1, Yes-associated protein1; ↑, increased or promoted effect; ↓, decreased or inhibited effect.

## MAMP-driven ISC modulation

MAMPs are molecules conserved in a variety of microbes, including commensals and pathogens, such as LPS, peptidoglycan, bacterial lipoprotein, CpG DNA motifs, and flagellin. These structures are not found in eukaryotic host cells and play a critical role in conferring innate and adaptive immunity.^104^ These MAMPs are sensed and recognized by PRRs, including TLRs and NLRs, which are primarily expressed by antigen-presenting cells (APCs) and epithelial cells. MAMP-PRR interactions have also been implicated in shaping the phenotypic and functional characteristics of ISCs (Figure 3).^7^

**TLRs:** TLRs are a class of evolutionarily conserved type I transmembrane proteins that include an ectodomain, transmembrane regions, and the cytoplasmic Toll-interleukin (IL)-1 receptor (TIR) domains. While the ectodomain with leucine-rich repeats (LRRs) recognizes MAMPs, TIR domains activate downstream signaling pathways.^105^ These TLR-mediated pathways operate through either the myeloid differentiation primary response 88 (MyD88)-dependent or MyD88-independent signaling routes. Activation of these pathways stimulates key transcriptional regulators, including NF-κB and MAPK, ultimately leading to the production of proinflammatory cytokines, chemokines, and antimicrobial peptides.^105^ TLR-MAMP interactions maintain intestinal homeostasis by regulating healthy epithelial barrier, epithelial regeneration, immune tolerance, and host innate defense.^94^ ^,^ ^105^ ^,^ ^106^ To visualize the expression of individual TLRs on IECs, Price et al. generated five strains of reporter mice. They found no specific expression of TLR2, 4, 5, 7 or 9 on ISCs, yet there was unexpected diversity in the temporal and spatial patterns of TLR expression on the IECs, governing their responsiveness to microbial stimuli (Price et al. 2018). Contrarily, several other studies point out an essential role for TLRs, particularly TLR4 and TLR2, in regulating the functional roles of ISCs.^107^ ^,^ ^94^ ^,^ ^108-112^ Neal et al. demonstrated through multicolor flow cytometry and fluorescent *in situ* hybridization that TLR4 is constitutively expressed in mouse intestinal crypts and on Lgr5+ ISCs.^110^ Overexpressing TLR4 in mouse intestinal epithelium leads to increased Lgr5+ stem cells and proliferation.^113^ In Contrast, LPS purified from the gut microbiota significantly reduced intestinal organoid growth, which was primarily composed of Lgr5+ ISCs.^109^ Furthermore, injecting mice with LPS not only enhanced apoptosis within the intestinal crypts, marked by increased expression of cleaved caspase-3, but also reduced crypt cell proliferation, evidenced by decreased incorporation of the nucleotide analog BrdU.^110^ TLR4^−/−^ mice, however, did not reflect these effects, which confirmed that LPS-induced intestinal crypt apoptosis and regeneration are dependent on TLR4 signaling.^110^ In line with this, targeted deletion of TLR4 from Lgr5+ ISCs inhibited LPS-induced apoptosis and proliferation. To understand how TLR4-LPS signaling executes these functions, wild-type (WT) and PUMA (p53 up-regulated modulator of apoptosis)^−/−^ mice were injected with saline or LPS. After LPS treatment, WT mice showed more apoptosis and less proliferation, but PUMA^−/−^ mice did not. This shows that PUMA plays a role in TLR4-mediated control of crypt apoptosis and proliferation. Further, induction of PUMA by TLR4 in intestinal crypts was dependent on TRIF but not MyD88. Importantly, PUMA ablation prevented mice from developing necrotizing enterocolitis (NEC) and reversed negative effects on crypt apoptosis and proliferation.^110^ Afrazi et al. showed that TLR4 causes endoplasmic reticulum (ER) stress, which promotes Lgr5+ ISC apoptosis, implying that increased ER stress in the neonatal intestine contributes to NEC susceptibility.^107^ Thus, selective targeting of TLR4-LPS interactions may be a therapeutic avenue to explore in the treatment of inflammatory diseases and injuries in the intestine.

TLR2 has been implicated in facilitating the regeneration of ISCs.^114^ ^,^ ^115^ ^,^ ^94^ ^,^ ^108^ ^,^ ^111^ Oral gavage with *Lactobacillus rhamnosus* GG (LGG) failed to protect intestine against radiation in TLR2^−/−^ or cyclooxygenase-2 (COX-2)^−/−^ mice, implying that LGG radioprotection involves signaling via TLR2 and COX-2 dependent prostaglandin E2 (PGE2) production.^114^ TLR2 has also been shown to induce regeneration of ISC niche cells, such as Paneth and goblet cells in the intestine.^108^ In addition, CL429, a TLR2 and NOD2 agonist conferred protection against radiation-induced intestinal damage in mice, which was dependent upon the activation of the TLR2-MyD88-NF-κB signaling pathway.^106^ A recent study by Fang et al. has thoroughly investigated the effects of CL429-TLR2 signaling and its interactions with other signaling pathways in the regulation of ISCs.^94^ After radiation exposure, CL429-treated mice had more BrdU+ cells, Lgr5+ ISCs, and Olfm4+ ISCs in the intestine compared to control mice. Irradiation of intestinal organoids from Lgr5-EGFP-IRES-creERT2 mice resulted in a significantly lower number of Lgr5+ ISCs, whereas treatment with CL429 significantly increased their abundance, indicating that CL429 protects against radiation injury by regenerating ISCs. Finally, TLR2^−/−^ mice exhibited reduced CL429 activation on YAP1, supporting an important role for CL429 in promoting ISC regeneration by activating TLR2-YAP1.^94^

**NLRs:** NLRs constitute a unique family of intracellular cytosolic receptors, including NOD1 and NOD2, which sense MAMPs and regulate host innate immunity.^116^ NOD2 is an important member of the NLR family that acts as a receptor for MDP, a conserved minimal motif of bacterial peptidoglycan, and plays important roles in host defense, autoimmunity, and autophagy.^117^ ^,^ ^118^ Studies have shown that NOD2 is constitutively and uniquely expressed in ISCs compared to other intestinal epithelial cells.^119^ Using intestinal organoids, Nigro et al. found that stimulation with agonists for TLR2, TLR4, TLR5, TLR9, or NOD1 had no effect on organoid growth. In contrast, selective activation of NOD2 significantly increased organoid formation in terms of numbers, but not size. Moreover, cultures of Lgr5+ ISCs with or without Paneth cells, showed enhanced organoid growth following NOD2 agonist stimulation. Finally, NOD2^−/−^ mice treated with doxorubicin, an agent that induces oxidative stress and ISC injury, exhibited increased cell death, demonstrating that NOD2 signaling via MDP plays a cytoprotective role in maintaining ISC viability under stress conditions.^119^ By using VilCre-NOD2^fl/fl^ mice we have shown that NOD2 in intestinal epithelial cells, including ISCs, is indispensable for the *Lactiplantibacillus plantarum* WJL induced increased numbers of Ki-67+ proliferating cells in the small intestinal crypts. This was linked with longer small intestine villi and improved systemic growth of juvenile mice during chronic undernutrition.^13^ Levy et al. recently deciphered the mechanism of NOD2-mediated cytoprotection using intestinal organoids and knockout (KO) mice.^12^ Using an irradiation stress model of mouse intestinal organoids, they demonstrated that MDP treatment suppresses total and mitochondrial reactive oxygen species (ROS) within ISCs, which was associated with mitophagy induction. Furthermore, intestinal organoids derived from the mice knocked out for ATG16L1 or NOD2 failed to show cytoprotection driven by MDP. *In vivo* findings further confirmed this finding that induction of mitophagy takes place by MDP-NOD2 pathway.^12^ Likewise, in another study, NOD2^−/−^ mice showed significantly lower organoid formation after irradiation compared to WT mice. This suggests that NOD2 promotes crypt survival and intestinal epithelial regeneration after irradiation-induced damage.^120^ Overall, these results elucidate the NOD2-mediated mechanism of cytoprotection, which involves the removal of harmful excess ROS via mitophagy, initiated by the synchronized activation of NOD2 and ATG16L1 through an NF-κB-independent pathway.

Overall, rather than functioning as independent microbial-sensing pathways, TLR4, TLR2, and NOD2 form an interconnected network that helps determine the balance between intestinal inflammation, antimicrobial defense, and epithelial repair. During homeostasis, relatively controlled TLR2 signaling contributes to barrier integrity and microbial tolerance, whereas excessive or dysregulated TLR4 activation in the setting of barrier disruption or dysbiosis can promote LPS-driven NF-κB- and MAPK-dependent inflammatory signaling. TLR2 and NOD2 can also contribute to protective intestinal responses, including epithelial barrier function, antimicrobial defense, and tissue protection. Importantly, NOD2 signaling can modulate TLR-mediated inflammatory responses, providing a regulatory link between intracellular sensing of bacterial peptidoglycan-derived muramyl dipeptide and cell-surface recognition of microbial products. NOD2 is also expressed in Lgr5+ ISCs, where its activation has been shown to promote stem-cell protection and epithelial regeneration, directly linking microbial sensing to intestinal stem-cell function.^119^ During dysbiosis or infection, therefore, the biological outcome is likely to depend not on activation of a single receptor alone but on the strength, timing, cellular context, localization, and coordination of multiple pattern-recognition pathways. Disruption of this regulatory balance may promote persistent inflammatory signaling and impair epithelial recovery, whereas appropriately coordinated TLR and NOD2 signaling can support antimicrobial defense and maintenance of intestinal homeostasis.^121^ ^,^ ^122^

It is important to note that bacterial components can also regulate ISC function indirectly through immune and stromal cells, impacting on the stem cell niche. TLR or NOD signaling in macrophages, dendritic cells, innate lymphoid cells, T cells or mesenchymal cells may reshape the ISC niche through IL-22, IL-6, TNF, IL-10, PGE2, CXCL12, Wnt ligands, R-spondins and BMP antagonists. Although this represents a crucial aspect of microbial regulation of the ISC niche, a comprehensive discussion of these mechanisms is beyond the scope of the present review. Readers interested in this topic are referred to recent reviews that discuss immune-epithelial-stromal cross-talk and its role in ISC regulation in greater detail.^123^ ^,^ ^124^

**Figure 3.**
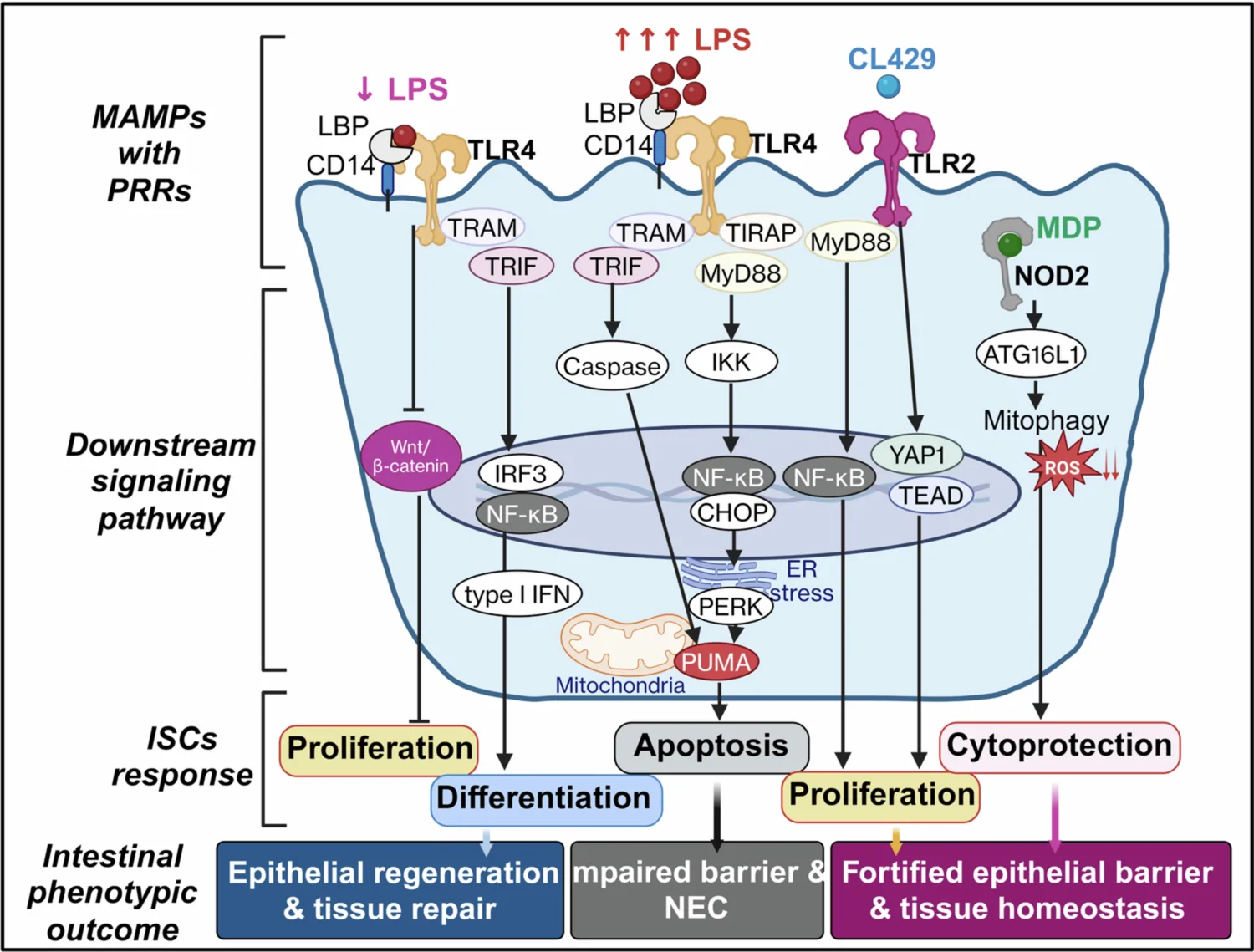
MAMP-PRR signaling regulates ISC responses and epithelial outcomes. Low (balanced) levels of LPS are recognized by the LBP-CD14-TLR4 complex and activates two major downstream signaling routes: the TRAM-TRIF-IRF3/type I interferon axis leading to ISC differentiation into epithelial lineages, including Paneth cells, and a Wnt/β-catenin-associated pathway that inhibits ISC proliferation. Overstimulation by high doses of LPS activates TLR4-associated TRIF/caspase signaling and the TIRAP-MyD88-IKK/NF-κB axis. The figure depicts caspase/PUMA-associated apoptosis and CHOP-PERK-PUMA signaling linked to ER and mitochondrial stress, alongside an NF-κB-associated proliferative response. These responses are associated with impaired epithelial barrier function and NEC. In contrast, stimulation of TLR2 by the receptor agonist CL429 activates MyD88-dependent or YAP1-TEAD signaling and promotes ISC proliferation, whereas recognition of MDP by NOD2 activates ATG16L1-dependent mitophagy, reduces ROS, and promotes ISC cytoprotection. MAMP-PRR signaling produces distinct ISC responses, including proliferation, differentiation, apoptosis, and cytoprotection, which are translated into tissue-level outcomes including epithelial regeneration and tissue repair, enhanced epithelial barrier integrity and intestinal homeostasis, or barrier impairment and NEC. Figure created with BioRender. Abbreviations: ATG16L1, autophagy-related protein 16-like 1; CD14, cluster of differentiation 14; CHOP, C/EBP homologous protein; ER, endoplasmic reticulum; IFN, interferon; IKK, IκB kinase; IRF3, interferon regulatory factor 3; ISC, intestinal stem cell; LBP, lipopolysaccharide-binding protein; LPS, lipopolysaccharide; MAMP, microbe-associated molecular pattern; MDP, muramyl dipeptide; MyD88, myeloid differentiation primary response 88; NEC, necrotizing enterocolitis; NF-κB, nuclear factor κB; NOD2, nucleotide-binding oligomerization domain-containing protein 2; PERK, protein kinase R-like endoplasmic reticulum kinase; PRR, pattern recognition receptor; PUMA, p53 upregulated modulator of apoptosis; ROS, reactive oxygen species; TLR2, toll-like receptor 2; TLR4, toll-like receptor 4; TRAM, TRIF-related adapter molecule; TIRAP, Toll-interleukin-1 receptor domain-containing adapter protein; TRIF, TIR-domain-containing adapter-inducing interferon-β; YAP1, yes-associated protein 1; TEAD, Transcriptional Enhanced Associate Domain.

## Microbiota-ISC interactions: implications for IBD and tumorigenesis

Although this review primarily focuses on microbiota-derived metabolites and MAMPs in the regulation of ISCs under steady-state conditions, these interactions may have broader implications for intestinal diseases in which epithelial homeostasis is disrupted. In particular, IBD is characterized by chronic mucosal inflammation, epithelial barrier dysfunction, and alterations in the composition and metabolic activity of the gut microbiota. Because ISCs continuously respond to signals from epithelial, immune, stromal, and microbial components of their niche, disease-associated changes in the intestinal microenvironment may alter the signals that normally regulate ISC proliferation and differentiation. Indeed, the microbiota-ISC niche is substantially remodeled during intestinal inflammation, with microbial metabolites and microbial-derived signals capable of influencing epithelial regeneration both directly and indirectly through other components of the niche.^125^

The mechanisms discussed throughout this review provide several potential links between microbial dysbiosis and impaired epithelial homeostasis in IBD. Alterations in microbial community composition can modify the production and availability of metabolites such as SCFAs, indole derivatives, lactate, and secondary bile acids, while disruption of the epithelial barrier may increase exposure of host cells to microbial products. These changes may affect ISC-associated signaling pathways, including Wnt/β-catenin, Notch, YAP, NF-κB, and receptor-mediated pathways such as TLR and NOD signaling. Importantly, microbial signals can exert opposing effects depending on their concentration, duration, cellular target, and metabolic context. For example, TLR2 and NOD2 signaling can contribute to epithelial protection and regeneration, whereas excessive TLR4 activation by LPS can promote ISC apoptosis and reduce crypt proliferation through a PUMA-dependent mechanism.^12^ ^,^ ^110^ Thus, dysbiosis may not simply increase or decrease ISC activity; rather, it may disturb the balance and timing of microbial signals required for appropriate epithelial renewal. This concept is consistent with evidence that the ISC niche undergoes substantial remodeling during intestinal inflammation and that altered microbial signals can influence both epithelial and non-epithelial components of this niche.^125^

Microbiota-ISC interactions may also be relevant to intestinal tumorigenesis. ISC-associated pathways such as Wnt/β-catenin, Notch, EGFR, and YAP are essential for physiological epithelial renewal but can contribute to abnormal proliferation when persistently or constitutively activated. Because Wnt signaling is a central determinant of ISC maintenance and is frequently dysregulated during colorectal tumorigenesis, alterations in microbial and inflammatory signals that converge on this pathway may influence the behavior of genetically altered epithelial populations.^126^ In addition, chronic inflammation can create an environment characterized by increased epithelial turnover, inflammatory signaling, and cellular stress, potentially favoring the expansion of cells carrying oncogenic alterations. Emerging evidence further suggests that specific bacteria can interact with stem-like epithelial or colorectal cancer stem-cell populations. For example, *Fusobacterium nucleatum* has been associated with colorectal cancer and has been reported to influence stem-like properties and tumor-promoting signaling, although the extent to which these effects involve normal ISCs versus transformed or cancer stem cells remains an important area of investigation.^127^ Therefore, microbiota-associated effects on tumorigenesis should not be interpreted as a simple consequence of increased ISC proliferation, but rather as the result of interactions among microbial signals, epithelial genetic alterations, inflammatory pathways, and the surrounding tissue niche.

Overall, these observations suggest that the physiological mechanisms by which microbial metabolites and MAMPs regulate ISC function may become pathologically relevant when the microbial environment, epithelial barrier, or host signaling network is persistently altered. Importantly, the available evidence does not support categorizing individual microbial metabolites or MAMPs as uniformly beneficial or detrimental. Instead, their effects appear to depend on the intestinal compartment, concentration, duration of exposure, cellular metabolic state, and host genetic and inflammatory context. A better understanding of how these variables alter microbiota-ISC communication may help explain the transition from physiological epithelial maintenance to defective regeneration in IBD or abnormal epithelial proliferation during tumorigenesis. However, further studies are needed to determine whether changes in microbiota-ISC signaling are causal drivers of these diseases or consequences of the altered intestinal environment.

## Therapeutic tractability and safety of microbiota-ISC regenerative signals

Not all microbiota-ISC signals are equally amenable to therapeutic development. Defined metabolites and receptor-directed pathways offer greater control over dose and exposure than complex interventions such as fecal microbiota transplantation (FMT). Among emerging candidates, the IPA-PPARα-HMGCS2-BHB axis is particularly promising, as IPA-induced BHB production promotes Lgr5+ ISC expansion and mucosal repair in experimental colitis, as discussed in the Microbial conversion of dietary substrates section.^84^ ILA may also contribute to this pathway through microbial conversion to IPA and subsequent BHB production.^83^ These findings suggest that IPA or BHB may represent more defined regenerative interventions than whole-microbiota manipulation. It is important to note that both IPA and BHB have systemic metabolic effects, and their intestinal availability may be limited by absorption, degradation, and microbial metabolism, highlighting the need for controlled or localized delivery. BHB is therefore a promising regenerative effector but is not ISC-specific.

Receptor-directed approaches also have potential but require careful consideration of specificity and dose. The TGR5 agonists, including INT-777, can promote ISC-associated SRC-YAP signaling and epithelial regeneration, whereas excessive exposure to certain bile acids may impair epithelial growth.^54^ Similarly, AhR ligands can regulate ISC activity and epithelial repair, but their effects depend strongly on ligand identity, concentration, and cellular context.^52^ ^,^ ^78^ Thus, neither TGR5 nor AhR activation can currently be considered a uniformly regenerative strategy. Engineered bacteria could provide localized production of therapeutic metabolites, but variable colonization, genetic stability, ecological effects, dissemination, and containment remain important limitations.^128^ FMT and conventional probiotics are less precise because their effects on microbial composition and metabolite production are difficult to standardize.

Importantly, therapeutic stimulation of ISCs must be balanced against potential tumorigenic effects. Wnt/β-catenin, Notch, EGFR, and YAP/TAZ promote physiological regeneration but can also support pathological epithelial expansion when persistently activated.^129^ Chronic stimulation could therefore favor expansion of pre-existing oncogenic clones, particularly in colorectal cancer-prone or chronically inflamed tissues. Future interventions should consequently aim for transient, spatially restricted, and reversible regeneration rather than sustained ISC proliferation, with long-term assessment of clonal expansion, dysplasia, and tumor formation in appropriate models.

## Conclusion and future directions

The gut microbiota contributes to the regulation of intestinal stem-cell activity by providing metabolic and microbial signals that influence ISC proliferation, differentiation, survival, and epithelial renewal. As discussed in this review, microbial metabolites, including SCFAs, indole derivatives, lactate, and secondary bile acids, together with MAMPs recognized by PRRs, can modulate ISC behavior through pathways involving Wnt/β-catenin, AhR, YAP, GPCRs, TLRs, and NOD signaling. These interactions highlight the importance of microbial cues in maintaining intestinal epithelial homeostasis.

An important consideration is that microbial effects on ISCs are not uniform. Their biological consequences can vary with metabolite concentration, intestinal location, cellular metabolic state, receptor expression, and the surrounding niche. The contrasting effects reported for butyrate and different indole derivatives exemplify this complexity. In addition, increasing evidence for ISC heterogeneity and plasticity indicates that future studies should investigate how distinct ISC populations respond to microbial signals rather than relying exclusively on canonical markers such as Lgr5. Furthermore, microbial effects should be interpreted in the context of ISC heterogeneity and epithelial cellular states. Active Lgr5+ ISCs, reserve HOPX/BMI1/LRIG1+ populations, TA cells, and niche cells may differ in their responsiveness to microbial metabolites and MAMPs, particularly during colitis, irradiation-induced injury, and organoid regeneration; therefore, responses attributed broadly to “ISCs” may reflect distinct cell-type-specific effects and dynamic interconversion between ISC populations.

It is also important to recognize that microbial dysbiosis may influence ISC function and intestinal epithelial repair. Changes in microbial community composition and metabolic activity modify host-microbiota interactions, alter the production and availability of microbiota-derived metabolites, and promote inflammatory and barrier-disruptive signals within the intestinal microenvironment. The gut microbiota has been shown to regulate ISC proliferation, differentiation, and regenerative capacity through direct microbial interactions as well as through immune, metabolic, and niche-mediated mechanisms involving pathways such as Wnt/β-catenin and Notch.^6^ ^,^ ^7^ Dysbiosis may therefore shift the intestinal environment towards an imbalance between microbial metabolites and signals that support epithelial repair and those associated with impaired epithelial homeostasis. On the other hand, specific commensal bacteria may enhance epithelial regeneration; for example, *Lactobacillus reuteri* has been reported to promote intestinal epithelial proliferation through R-spondin-dependent activation of Wnt/β-catenin signaling.^130^ Collectively, these findings suggest that microbial dysbiosis should be considered an important confounding factor when evaluating the effects of microbiota-derived tryptophan metabolites on ISCs, because alterations in microbial community structure may affect both metabolite availability and the responsiveness of the intestinal epithelial niche.^131^ These microbiota-ISC interactions may also have broader implications for intestinal disease. As discussed above, alterations in microbial composition and signaling may disturb epithelial regeneration and inflammatory homeostasis in IBD and may influence pathways involved in intestinal tumorigenesis. Nevertheless, further studies are needed to determine whether altered microbiota-ISC signaling is a primary driver of disease or a consequence of the inflammatory and tissue environment.

Future research combining single-cell and spatial approaches with metabolomics, lineage tracing, organoid models, and defined microbial systems should help clarify which microbial signals act directly on ISCs and which influence them indirectly through epithelial, immune, or stromal cells. Such mechanistic understanding could support the development of targeted microbiota-based interventions, including dietary strategies, probiotics, postbiotics, and microbial metabolites, to enhance epithelial resilience while limiting undesirable inflammatory or proliferative responses.

## Data Availability

No datasets were generated.
